# LOLATAO—An Artificial-Intelligence-Based Virtual Assistant for Clinical Follow-Up of Patients with Non-Valvular Atrial Fibrillation (AF) Undergoing Oral Anticoagulant Therapy (OAT): A Feasibility Study

**DOI:** 10.3390/jcm14093023

**Published:** 2025-04-27

**Authors:** Amparo Santamaría, Cristina Antón-Maldonado, Beatriz Sánchez-Quiñones, Nataly Ibarra Vega, Pedro González, Rafael Carrasco

**Affiliations:** 1 Hybrid Hematology Department, University Hospital Vinalopó, 03293 Alicante, Spain; camaldonado@vinaloposalud.com (C.A.-M.); bsquinones@vinaloposalud.com (B.S.-Q.); niibarra@vinaloposalud.com (N.I.V.); 2 Fundación Para el Fomento de la Investigación Sanitaria y Biomédica (FISABIO), 46020 Valencia, Spain; 3 Management Department, University Vinalopó Hospital, 03023 Alicante, Spain; pgcabezas@vinaloposalud.com (P.G.); rcarrasco@vinaloposalud.com (R.C.)

**Keywords:** virtual assistant, artificial intelligence, oral anticoagulation, atrial fibrillation

## Abstract

**Background:** The aim of this study was to evaluate, for the first time, the feasibility of implementing LOLA, a speech-AI-driven conversational assistant, in monitoring and managing OAT for patients with non-valvular atrial fibrillation (AF). **Methods:** In 2023, we conducted a pilot prospective observational study of patients with non-valvular atrial fibrillation (AF) and TAO. All patients received a first-contact call from LOLATAO and then monthly calls following a protocol predefined by haematologists. At the end of the study, a satisfaction questionnaire was carried out. **Results:** Of the fifty patients, the mean age was 75 years, and 33% were women. One-third of the patients (n = 16) were receiving antivitamin K treatment, and two-thirds (33) DOACs. A total of 579 calls were made with a median follow-up of 278 days. LOLATAO had high rates of acceptability (85%), adherence (90%), and satisfaction (>95%). A total of 42% of the patients reported at least one missed dose within the last month, and 18% reported having a scheduled intervention requiring bridging therapy. In patients with AVKs, 94% (n = 15) reported being unaware of their TRT at least once, and 75% (n = 12) of patients reported having a TRT < 65%. Those patients in whom the TRT was <65% were switched to DOACs. LOLATAO saved a total of 10 h per month for haematologists during follow-up. **Conclusions:** This study suggests that LOLATAO can be a helpful tool in the management of chronic follow-up of patients with AF and undergoing OAT, reducing the burden of care and with high rates of acceptance and satisfaction by patients.

## 1. Introduction

Non-valvular atrial fibrillation (AF) is the most frequent arrhythmia in adults, and its prevalence is expected to double in the next 30 years [1,2,3]. Also, AF increases the risk of stroke five-fold [4]. Oral anticoagulation therapy (OAT) decreases the risk of cardioembolic stroke and all-cause mortality in patients with AF [5,6,7,8,9,10]. Thus, OAT is the gold standard for the prophylaxis of AF-related stroke [6,7,8]. Effective OAT implies a lifelong clinical follow-up, including regular assessments of bleeding risk, drug interactions, side effects, dosing adjustments, and control of adherence. Clinical guidelines advocate for a patient-centric and multidisciplinary approach to integrated AF care. This approach emphasises structured follow-up intervals, ranging from every 1 to 6 months, tailored to individual patient factors [6,7,8,11].

The growing healthcare burden associated with AF poses challenges for healthcare centres in implementing this comprehensive care model. Digital health tools facilitate coordination among various specialties (such as cardiologists, haematologists, general practitioners, and nurse-led teams) and enhance patient engagement through home-based services [8,12,13]. Multiple e-health solutions have already been used in AF across all stages of the disease with promising results, from screening and diagnosis through mobile or wearable ECG devices [14,15,16,17,18] to computerised decision-making support systems for antithrombotic therapy optimisation [19,20,21,22] and point-of-care monitoring of anticoagulation for patients using AVKs [23,24,25], as well as mobile health (mHealth) interventions to increase treatment adherence [26,27], manage cardiovascular modifiable risk factors [22,28,29,30,31], and improve patients’ disease literacy [22,26,27,32].

Conversational agents are a type of mHealth intervention that facilitate automated telephone communication with patients and can therefore enhance healthcare accessibility and improve the reach of clinical services over face-to-face consultation [33,34,35,36,37]. Because of this heterogeneity, it is very difficult to establish a standardised method for evaluating conversational agents in the healthcare setting. That is why results from the studies evaluating conversational agents have shown promising but inconsistent effects on their accuracy, user acceptability and effectiveness. Most of the healthcare conversational agents reported in the literature were predominantly unidirectional and non-AI-driven [33,34,35] and those incorporating AI technologies were mostly text-based even though speech interactions seem to be more natural and comfortable [36,37]. There are few examples of speech-AI-driven virtual assistants in medicine. The importance of these systems falls on its understanding of what the user says (speech recognition), the ability to plan an appropriate reaction (natural language processing), and articulating the response (natural language generation) [38,39,40,41].

Nevertheless, voice assistants like LOLA from TUCUVI focus more on customisation and adaptation to the specific needs of the user in different clinical contexts. These medical assistants can significantly aid in reducing the workload of healthcare professionals and enhancing patient care efficiency.

Despite this progress, our literature search reveals a critical gap, no AI-driven automated telephone interventions exist for patients with AF or for monitoring anticoagulation treatment [30,31,32,33,42,43,44,45]. Our study aims to address this void and evaluate the potential impact of AI-driven conversational agents in these specific contexts.

Given the existing evidence, the aim of this study was to evaluate, for the first time, the feasibility of implementing LOLATAO, a speech-AI-driven conversational assistant, in monitoring and managing OAT in patients with AF.

## 2. Material and Methods

### 2.1. Study Design and Population

From 1 January 2023 to 31 December 2023, we conducted an observational prospective study in the Anticoagulation Unit of our Digital Unit [46,47] at the University Vinalopó Hospital. Approval was obtained from the local ethics committee. (date: 22 October 2022; code ID: LOLA_TAO). Patients were recruited consecutively from our ambulatory facilities. The inclusion criteria included the following: (1) aged ≥18 years; (2) a documented diagnosis of non-valvular AF; (3) receiving OAT; (4) had a mobile phone that was able to receive calls; and (5) were competent in the Spanish language. Participants were excluded from the study if they (1) had a medical illness with anticipated life expectancy of <6 months, (2) were unable to provide written consent, or (3) had a concomitant illness or physical impairment (for example, hearing impairment) or mental condition that, in the opinion of the study team or the primary physician, could interfere with the conduct of the study, including outcome assessment.

**Figure 1:**
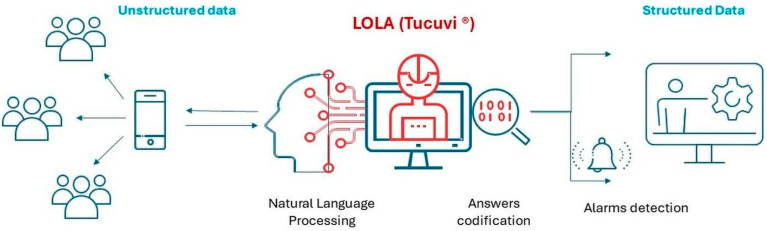
LOLATAO at a glance.

The study was based on the use of an AI-based voice virtual assistant (LOLA), a medical device from the company TUCUVI Care SL, (version 3.37.12, Tucuvi Care S.L C/ Hermosilla 48, 1D, 28001, Madrid, Spain). It is a voiced-based conversational technology that performs AI-driven automated phone calls. This technology simulates human conversations with proper responses to dialogue. LOLA can analyse verbal speech using VRS and NPL. Then, it uses a speech-to-text algorithm to obtain and codify data in a structured database (See Figure 1).

The protocol study’s workflow, chronogram, and the various protocols designed for the OAT treatment are presented in Supplementary Materials Figure S1.

First, the medical team established a protocol of questions and possible answers for each type of OAT and defined which answers to interpret as an “alert” by the virtual assistant. (See Supplementary Materials Table S1). Then, regular screening of upcoming patient appointments was conducted by the research team to identify potential participants over a 6-month period (from January to June 2023). These participants were approached (1) face-to-face during their visit to obtain written consent or (2) over the telephone before they attended their scheduled appointment. If they met the inclusion criteria, a research staff member arranged a personal meeting with the prospective participants. All interested participants received an information sheet and a consent form describing the study and providing sufficient information for the participant to decide whether to participate in the study. Afterward, the consent form was signed by both the participant and the research staff before the participant undertook any study procedure. Eligible individuals who provided informed consent were asked to complete a baseline assessment, and demographic data were collected. After the baseline data collection, patients were registered in the TUCUVI Dashboard, an AI web platform (See Supplementary Materials Figure S2), and were assigned a predefined protocol according to the type of OAT. Patients were recruited consecutively in our ambulatory facilities. While our study population consisted of relatively healthy patients with AF, this allowed us to focus on the impact of LOLATAO on adherence and monitoring in a population where these factors are critical for preventing complications. Demographic characteristics and type of OAT treatment are in Table 1.

Once the patients were included in the web platform, outreach by LOLA occurred within 24 to 48 h from the baseline assessment to welcome the patient to the study and inform them about the follow-up process (i.e., initial phone call) and then monthly until the end of the 12-month follow-up period (i.e., follow-up phone calls). The definition of “alert” was established by the medical team prior to the study’s initiation. Specific conditions/events that triggered an alert included patient responses indicating missed medication doses, reports of bleeding events, need for bridging therapy, impaired renal function, unknown therapeutic range time (TRT), and TRT below 65%. These criteria were detailed in a protocol of questions and possible answers, with each answer mapped to whether it should generate an alert by the virtual assistant (see Supplementary Materials Table S1). When LOLA detected that an answer fulfilled the definition of an alert, a notification was sent to the physician in charge of the patient. Then, the physician reviewed the answer and medical history of the patient and decided whether to perform an intervention. At the end of the study, the patients received a phone call with a satisfaction questionnaire (final phone call).

The primary outcome of the study was to assess the acceptability, adherence, and satisfaction of implementing LOLA, an AI-based conversational agent, to follow-up with patients with AF undergoing OAT. The secondary outcomes were to analyse the usability and clinical utility of LOLA. A summary of the parameters evaluated is provided in Table 2. Acceptability was evaluated by measuring the percentage of patients that signed the informed consent form by the total number of eligible patients. During follow-up, adherence to the program was evaluated monthly through program engagement metrics (e.g., number of answered telephone calls and number of completed telephone calls). Patient usability was evaluated by measuring the duration of each phone call, and the usability by medical doctors was measured by the number of alerts reviewed and interventions performed. The physician in charge was automatically notified of alerts detected by the AI-based virtual assistant (i.e., LOLA). The physician then reviewed the alert and the patient’s medical history to determine whether an intervention was necessary. Interventions were not performed automatically by the AI; they required active review and action by a medical doctor, which could include direct phone calls or in-person communication with the patient. Intervention and alert use metrics were automatically collected through the AI-based conversational agent system and website analytics. Clinical outcomes were evaluated monthly by measuring the number of clinical situations detected, the concordance between the alerts detected and the clinical condition referred by the patient, and the number of hours that the medical team saved by use of the AI-conversational agent system. Clinical situations detected were classified as follows: medication adherence (self-reported), health events (self-reported and medical records), and healthcare service use (self-reported and medical records). The medical team revised the concordance between the alerts detected and the medical records/patient contact to assess whether the alerts detected were related to actual clinical events. The number of hours the medical team saved by LOLATAO was calculated automatically using a formula based on total calls made to the patients and the time that the doctor would have needed to conduct a follow-up call. To calculate the time spent by a doctor vs. LOLATAO, it was considered that LOLATAO is 20% more efficient than a human being in terms of follow-up visits. On the other hand, the time it would take a person to prepare for the call is also taken into account (almost 2 min). Satisfaction was evaluated at the end of the study via a study-specific questionnaire, net promoter score (NPS), and customer satisfaction (CSAT), also assessed through LOLATAO [48,49].

### 2.2. Statistical Methods 

We calculated the descriptive statistics, including means, medians, and ranges. Although the sample size was insufficient for meaningful regression analysis, we also tested for differences across age and gender groups using 2-tailed *t*-tests and chi-square tests. The power for these comparisons was low; therefore, this analysis was an exploratory evaluation that may be important for future study design.

## 3. Results

This pilot study enrolled a cohort of 50 patients, of which one was excluded posthumously from the analysis. The mean age was 75 ± 16 years old, and most of them were male (33% women). Regarding OAT, one-third of patients were on AVKs (n = 16), and two-thirds on direct oral anticoagulants (DOACs) (n = 33) (See Table 1).

**Table 1:** Demographic characteristics and type of OAT treatment.

Demographics						
Age (mean, range) = 75 ± 16	AVKs (n = 16)		DOACs (n = 33)		Total (n = 49)	
	Male n = 12	Female n = 4	Male n = 29	Female n = 11	Male n = 33	Female n = 16
Type of DOAC (n = 33)						
Edoxaban (n, %)	7 (21%)					
Rivaroxaban (n, %)	11 (33%)					
Apixaban (n, %)	12 (36%)					
Dabigatran (n, %)	3 (9%)					

**Figure 2:**
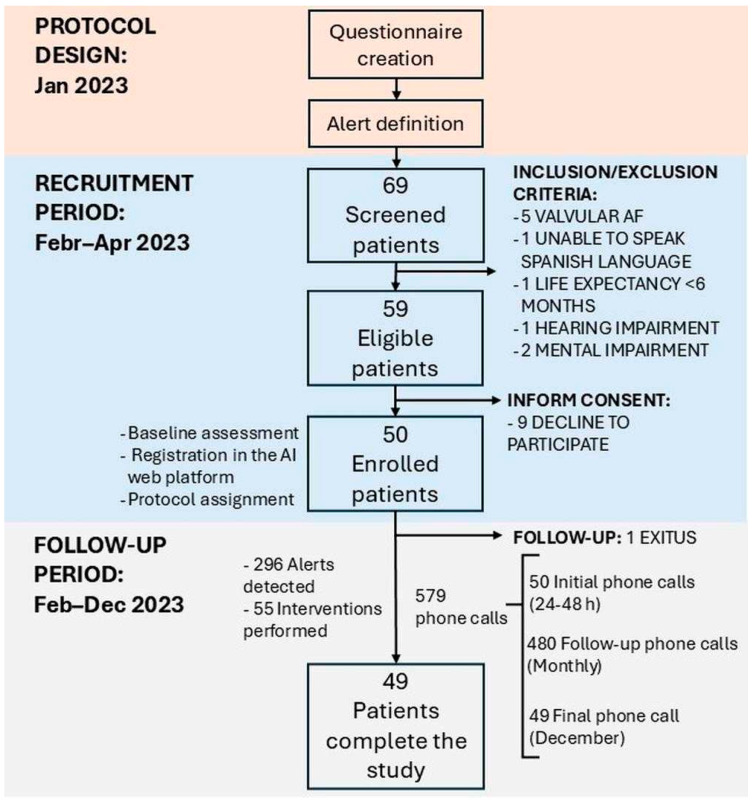
Workflow of the Study.

The workflow during the study is continued in Figure 2. Oral anticoagulant therapy was administered, with a division of one-third on AVKs and two-thirds on DOACs. Throughout a median follow-up period of 278 days, a total of 579 telephone calls were conducted, comprising 50 initial, 480 follow-up, and 49 final calls, with an average duration of 3 min and 17 s per call (range: 3.05–3.81). During the study, 296 alerts were detected, which led to 55 interventions by the medical team. At the end of the study, the team saved more than 110 h/year by using LOLATAO.

LOLATAO demonstrated high acceptability at 85% and adherence at 90%, maintaining these rates consistently over the follow-up period. Despite the number of alerts remaining constant, the frequency of interventions decreased, with only 19% of alerts necessitating an intervention. Clinical outcomes revealed that approximately 40% of patients reported missing at least one dose in the previous month, with no significant difference between AVKs and DOACs. The accuracy of the self-reported missed doses was based solely on patient recollection during AI-driven calls. There was no independent verification (such as pill counts or electronic monitoring) of adherence; the system relied on patient responses to structured questions, and the adherence data were self-reported and not independently validated. The clinical issues identified included bleeding events, the necessity for bridging therapy, renal function impairment, unknown therapeutic range time (TRT), and TRT below 65%, with the latter two showing lower concordance rates of 57% and 65%, respectively. It is noteworthy that over 90% of patients on AVKs were initially unaware of their TRT, but after education, only a quarter of those who reported a TRT below 65% actually had such rates. It is important to point out that the INR interval typically followed clinical practice guidelines (every 4–6 weeks or as clinically indicated), which is the typical INR monitoring interval, and to specify that the LOLATAO calls were scheduled monthly and not necessarily aligned with INR testing dates (see Table 2). Regarding renal impairment, 40% of the patients were already affected before starting the study, yet none required dose adjustments during the follow-up. Bleeding events occurred in 40% of the cohort within the last month and more frequently among those on AVKs, although all incidents were minor. About 20% of patients had scheduled surgical interventions within the forthcoming month, with bridging therapy being more prevalent among those on DOACs due to users of AVKs planning their surgical procedures during the INR control visits. In terms of healthcare service utilisation, 36% of patients visited the emergency department, and 6% required hospitalisation, none of which were related to OAT. The LOLATAO showed high levels of acceptability and adherence, which are further evidenced by the 85% participation rate among eligible patients, with more than 90% of follow-up calls answered and completed. The stability of the alert numbers contrasted with a decline in interventions, as only 19% of detected alerts led to an intervention (see Supplementary Materials Figure S3).

**Table 2:** Patient and clinical outcomes. (NA: not available).

OAT Adherence				
Medication doses missed	AVKs (n = 16)	DOACs (n = 33)	Total (n = 49)	
0	10 (63%)	18 (55%)	28 (58%)	
1	4 (25%)	5 (15%)	9 (18%)	
2	1 (6%)	7 (21%)	8 (16%)	
≥3	1 (6%)	3 (9%)	4 (8%)	
Health Events				Concordance (n, %)
≥1 Bleeding event	14 (88%)	5 (15%)	19 (39%)	19 (100%)
Bridging therapy	1 (6%)	8 (24%)	9 (18%)	9 (100%)
Renal function impairment (TFG < 60)	-	14 (42%)	NA	8 (57%)
Unknown TRT	15 (94%)	NA	NA	15 (100%)
TRT < 65%	12 (75%)	NA	NA	9 (75%)
Healthcare service use				
Emergency department	5 (31%)	12 (36%)	17 (35%)	17 (100%)
Hospitalisation	0 (0%)	3 (9%)	3 (6%)	3 (100%)

Initially, the agreement between LOLATAO and the alerts was low, but as a pilot study, as time progressed, it improved as the assistant was trained to better discriminate alerts, and the medical team adjusted the alerts accordingly. By the end of the study, the concordance was high enough that few interventions or unscheduled visits by the haematologist team were required. Adherence data from the 480 follow-up calls indicated that up to 92% responded to the calls, and 91% completed them. The survey showed a high level of satisfaction, as measured by the NPS and CSAT (see Table 3).

We observed a potential cost-savings associated with the 110 h/year saved by the haematologists, such as redeployment of staff or increased patient throughput. But we also acknowledged that a full cost-effectiveness analysis was not conducted and suggests that future studies should include a formal economic evaluation of LOLA’s implementation.

## 4. Discussion

To the best of our knowledge, this is the first evaluation of an AI conversational technology to support AF anticoagulation-integrated care, and our findings contribute to the growing body of research on the use of AI in healthcare. Our findings indicate the achievement of the primary goal of this study, demonstrating the practicality of deploying an AI-conversational agent for monitoring OAT in patients with AF within a hybrid haematology unit [46]. This aligns with our strategic trajectory of digital transformation, where telemedicine has been successfully integrated into the management of anticoagulated patients in the Anticoagulation Unit [47]. It is noteworthy that LOLATAO enables a more thorough follow-up compared to traditional methods, ensuring higher levels of engagement and adherence. It liberates physicians from the time-intensive follow-up process, allowing for prioritisation of care for those in need over stable patients. With only 37% of calls triggering an alert but only 19% needing an intervention, over 60% required no review, enabling us to discern critical alarms and necessitate a telematic visit, thus reducing physician calls by over 60% and, consequently, the healthcare burden.

This investigation presents data on the viability of an AI-conversational agent for the standard follow-up of OAT in patients with AF. It provides an assessment of the implementation process, acceptability, adherence, satisfaction, and clinical value of this digital healthcare intervention. This study addresses a gap in the knowledge regarding the role of AI-conversational automated monitoring in aiding patients with chronic conditions and establishes a customised follow-up plan tailored to the attributes of each patient. Additionally, it examines the potential advantages and limitations of the AI system for AVKs versus DOACs. For example, the AI system may be particularly beneficial for patients on AVKs who require more frequent monitoring and dose adjustments. The finding that the AI system improved TRT awareness in patients with AVKs supports this. However, the AI system can also enhance adherence and identify potential issues in patients on DOACs, who may be less frequently monitored in routine practice.

Presently, various virtual assistants exist, each underpinned by distinct machine learning models. For instance, LOLATAO has been documented as being used to track COVID-19 patients. A review of clinical trials reveals that merely five studies meet the specified eligibility criteria, yet none resemble the virtual assistant LOLA [39,40,41,42,43,44,45].

The study also addresses critical issues concerning the adherence and educational deficit among patients undergoing OAT and concurs with extensive research on this topic [50]. Clinically, LOLATAO has underscored the need for diverse healthcare professionals and clinicians to educate patients about the importance of adherence, even post-development of any safety or efficacy outcomes. In conventional anticoagulation units, the high demand for care makes such comprehensive, monthly patient monitoring challenging [50]. For this reason, the haematologist team played a crucial role in the implementation of LOLATAO in the OAT unit. LOLATAO needs to be programmed to obtain better results, so the investigational team—the haematologists—contributed to the process with their clinical expertise, providing the essential medical knowledge required to program and fine-tune LOLATAO. Therefore, they play crucial roles in the implementation and operation of LOLATAO, ensuring that the system is clinically effective, patient-focused, and continuously improving. Their role encompasses both the technical aspects of programming and adjusting the AI system and the human aspects of patient care and education. After the pilot study, LOLATAO is now ready to be implemented in other anticoagulation units.

Regarding the significance of the study, it is noteworthy that the results highlight the promising role of AI-based virtual assistants in enhancing patient management for AF in OAT, potentially leading to more tailored and efficient healthcare solutions. However, this study has several limitations, as follows: it was conducted at a single institution with a small cohort and brief follow-up period; it lacked a control group; and there was a gender imbalance among the participants. Further studies are required to evaluate LOLATAO in more complex patients with AF with multiple comorbidities in order to address potential selection bias and improve the generalisability of the findings.

Several implications of the study are that conversational AI interventions have the potential to augment clinical care by providing continuous patient support between visits. Future software advancements should aim to integrate such systems with existing electronic medical records in both primary and secondary care settings, representing a significant hurdle for the health information technology industry. Facilitating clinicians’ direct access to data gathered by the system, such as risk assessments and symptom evaluations, would mark a pivotal advancement in the integration of AF care.

Therefore, LOLATAO, as an AI-assistant in anticoagulation management, has proven through our pilot study to enable tighter control, reduce care demand without compromising quality, and provide personalised attention, all while achieving high patient satisfaction among anticoagulated individuals.

Also, the information obtained as a secondary objective of the study gave us information related to the practical value or usefulness of an artificial intelligence (AI)-driven assistant based on data collected during a preliminary study. Such studies are essential for assessing the actual clinical performance of AI systems, ensuring safety, evaluating human factors, and paving the way for larger-scale trials.

Artificial intelligence (AI) can significantly enhance healthcare outcomes for patients with AF through various means such as personalised care. For example, LOLATAO can conduct a vast number of patient phone calls, obtaining data and providing personalised interventions that lead to improved patient outcomes, as well as better effectivity in treatment because of the ability to automate outreach based on patient journeys, support clinical adherence and engaging patients effectively. Importantly, it supports the healthcare workforce by automating these tasks, meaning healthcare professionals can focus more on meaningful interactions with patients, positively affecting health outcomes.

In summary, the AI-driven assistant LOLATAO has the potential to transform the care experience for patients undergoing OAT by offering personalised, efficient, and integrated healthcare solutions. The use of LOLATAO in clinical practice may reduce the need for frequent monitoring and interventions, leading to cost savings and improved patient outcomes, saving patients multiple visits for health services. Further investigations are essential to evaluate the clinical impact of such interventions.

## Figures and Tables

**Table 3:** Results of the satisfaction surveys.

Questionnaire	Result	Explanation
CSAT	4.63/5	All patients, except 1, indicated being satisfied or very satisfied with LOLA; 1 patient answered with a rating of 3 (i.e., neutral).
NPS	44.73%	38 patients answered this question, and only 5 of them provided a rating lower than 7.

## Data Availability

The data supporting the findings of this study are available in the institutional repository of Vinalopó University Hospital, managed by the Office for Clinical Research Coordination (OCIC). Access to the data can be granted upon reasonable request and is subject to the hospital’s privacy and confidentiality policies. For further information, please contact the hospital’ Investigation Service through its website https://extranetsalud.vinaloposalud.com/ (accessed on 20 April 2025).

## Supplementary Materials

The following supporting information can be downloaded at: https://www.mdpi.com/article/10.3390/jcm14093023/s1.
