# Supplementary material for: LOLATAO—An Artificial-Intelligence-Based Virtual Assistant for Clinical Follow-Up of Patients with Non-Valvular Atrial Fibrillation (AF) Undergoing Oral Anticoagulant Therapy (OAT): A Feasibility Study
Source: J Clin Med. 2025 Apr 27;14(9):3023. doi: 10.3390/jcm14093023 (PMC12072667; doi:10.3390/jcm14093023)
Supplement: Supplementary file 1 [file jcm-14-03023-s001.zip › jcm-3532944-supplementary.pdf]

SUPPLEMENTAL FIGURE S1. TUCUVI HEALTH MANAGER AND LOLATAO

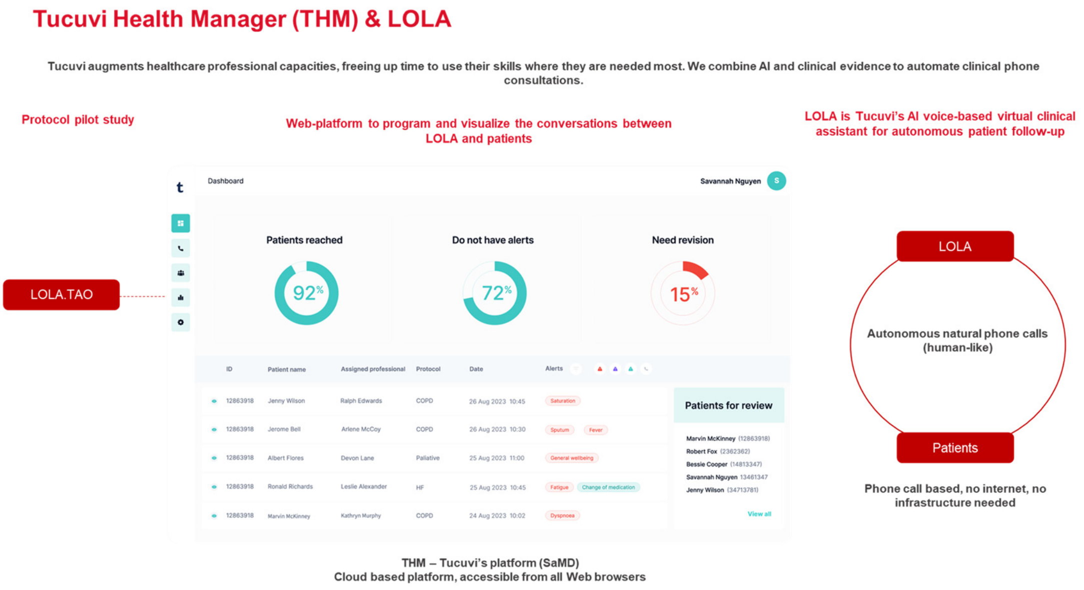

Supplemental Figure S2. Patient and Professional Flow in General and different protocol Flow depending on the type of OAT such as apixaban, rivaroxaban, dabigatran and edoxaban.

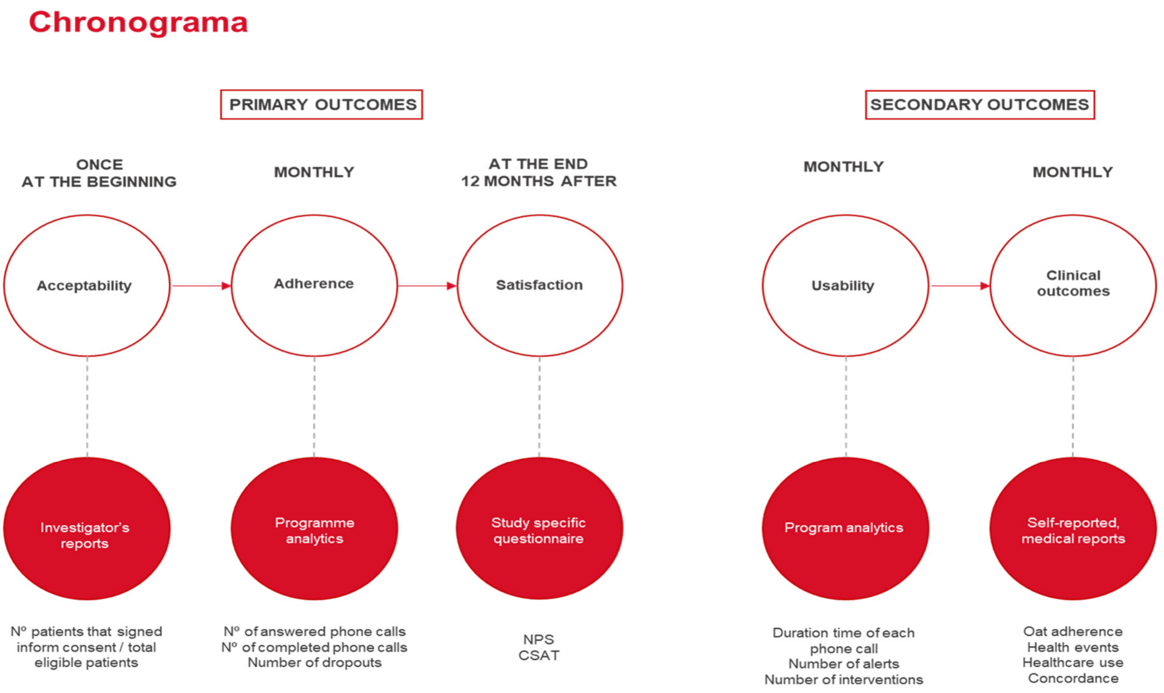

Patient and professional flow

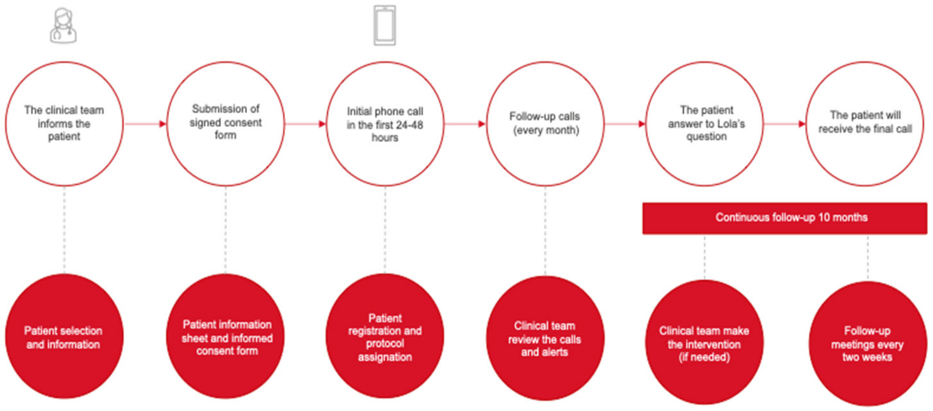

3

Apixaban protocol

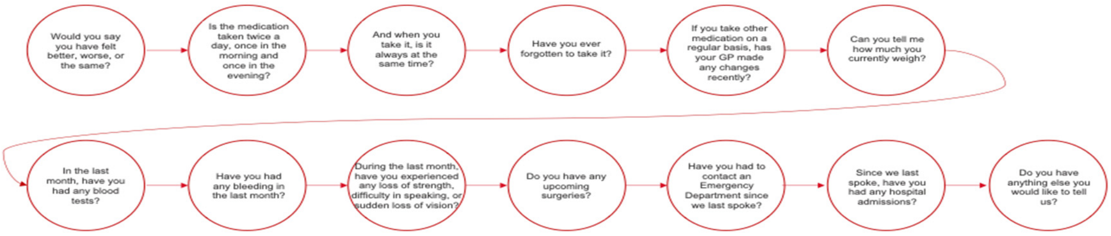

5

Ribaroxaban protocol

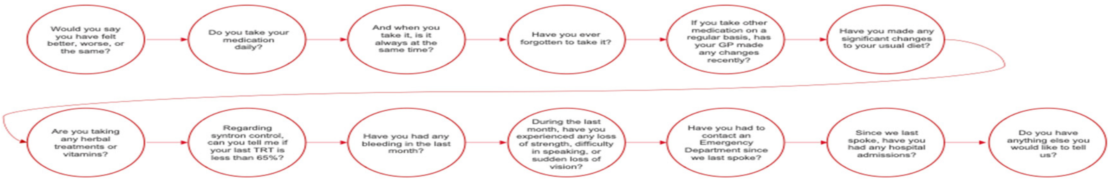

4

Acenocumarol protocol

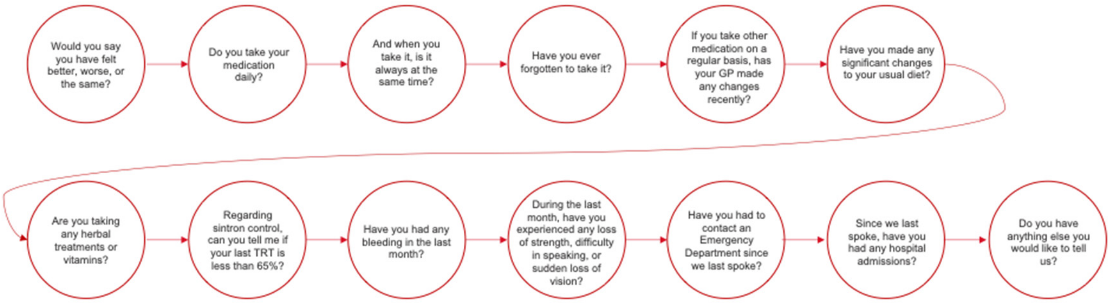

8

Warfarin protocol

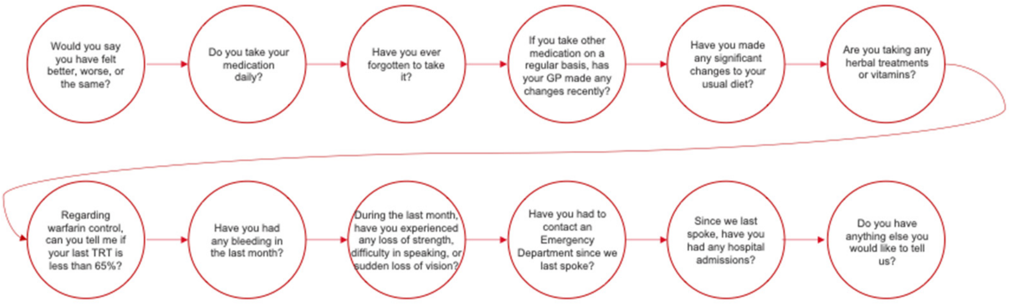

9

Final call protocol

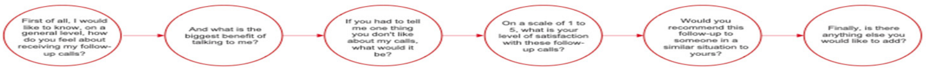

10

Supplemental Figure S3.: Alerts

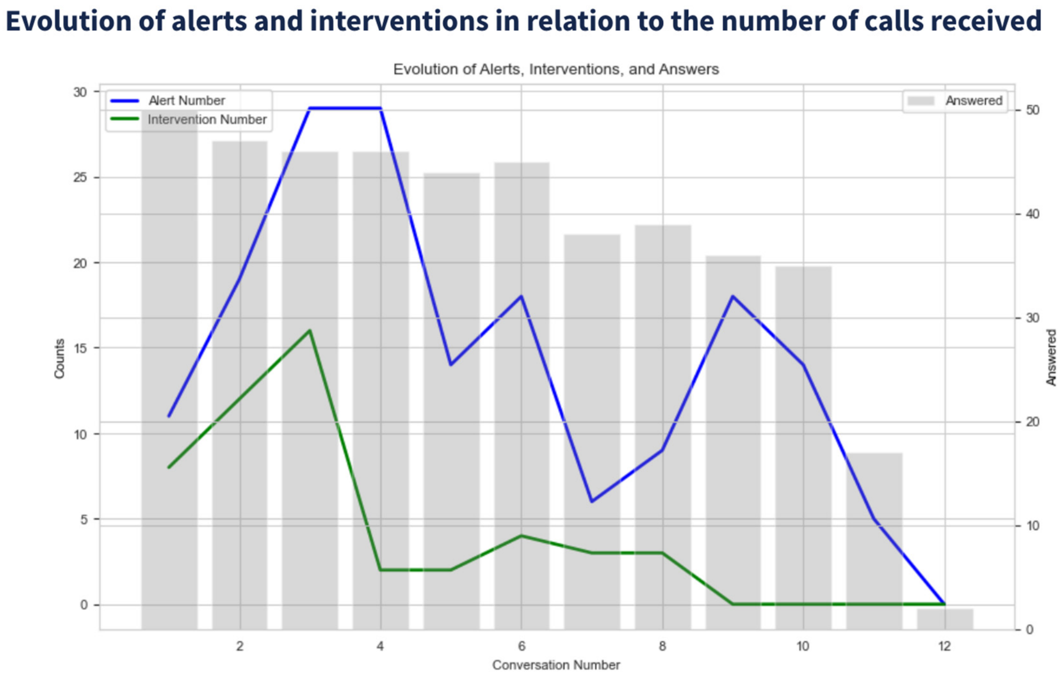

Supplemental Table S1. Description of the Scheme of Initial phone call (all patients) and Final phone call and creation of alerts depending on the Alert Criteria.

Initial Phone Call (All patients)

LOLA's question	Patient's answer	Alert criteria
Introduction and patient's identification: Hello, I'm Lola, the virtual clinical assistant of the Hematology Unit of the Vinalopó Hospital. I would like to speak with [patient name], is that you?	Pacient / Caregiver /	

Final phone call (All patients)

LOLA's question	Patient's answer	Alert Criteria
I would like to know; how do you feel about receiving my follow-up phone calls?	[Free answer]	
In your opinion, what do you think is the most beneficial part of talking to me?	[Free answer]	
If you had to choose a negative aspect of my calls, what would it be?	[Free answer]	
From 1 to 5, which is the level of satisfaction with this follow-up phone calls? Being 1 not satisfied at all and 5 very satisfied.	1,2,3,4,5	

Would you recommend this kind of follow-up to a person in the same situation as you?	No, I wouldn't recommend it / Yes, I would recommend it / Don't know	
Is there anything else you would like to add? Like, for example, things that in your opinion could be improved or any comments you want to make	No / Yes, [Free answer]	

Follow-up phone calls depending of the type of AOT:

Questions during Follow-up Phone Calls in the apixaban Protocol

LOLA's question	Patient's answer	Alert Criteria
Do you feel better, worst or the same as the last time we talked?	Same/ Better/ Worst	
Do you take your medication twice a day, one in the morning and the other one in the afternoon?	No / Yes	
When you take your medication, is it always at the same time?	No / Yes/ Don't know	
Did you forget to take your medication in the last month?	No / Yes, [number of times] / Don't know	

Did your primary care physician made any change in your chronic medications?	No / Yes / Don’t know / [Free answer]	Yes
Can you tell me how much do you weight?	[Weight] / Don’t know	<60.5 kilograms
Did you have a blood test performed in the last month?<br>If the answer is yes, was your kidney fine?	No/ Yes / Don’t know<br><br>Fine/ Bad / Don’t know	
Did you have any bleedings in the last month?<br>If the answer is yes, do you feel you need a medical consultation?	No / Yes, [number of times]/ Don’t know.<br><br>No/ Yes	Yes
Do you have any surgical intervention planned in the next month?<br>If the answer is yes, do you need a medical consultation?	Yes / No<br><br>Yes / No	Yes
Did you attend the emergency department since the last time that we talked?	No / Yes, [reason]	Yes
Did you have any hospitalization since the last time that we talked?	No / Yes, [reason]	Yes
Is there anything else you want to tell me?	No / Yes, [Free answer]	

Questions during Follow-up Phone Calls of Edoxaban, dabigatran,and rivaroxaban Protocol.

LOLA's question	Patient's answer	Alert Criteria
Do you feel better, worst or the same as the last time we talked?	Same/ Better/ Worst	
Do you take your medication every day?	No / Yes	
When you take your medication, is it always at the same time?	No / Yes/ Don't know	
Did you forget to take your medication in the last month?	No / Yes, [number of times] / Don't know	
Did your primary care physician made any change in your chronic medications?	No / Yes / Don't know / [Free answer]	Yes
Can you tell me how much do you weight?	[Weight] / Don't know	
Did you have a blood test performed in the last month?<br>If the answer is yes, was your kidney fine?	No/ Yes / Don't know<br>Fine/ Bad/ Don't know	 Bad / don't know
Did you have any bleedings in the last month?<br>If the answer is yes, do you feel you need a medical consultation?	No / Yes, [number of times]/ Don't know.<br>No/ Yes	Yes
Did you feel loss of strength, speaking difficulties or acute loss of vision in the last month?<br>If the answer is yes, did you seek medical attention?	No / Yes / Don't know.<br>Yes / No	Yes

Do you have any surgical intervention planned in the next month?<br>If the answer is yes, do you need a medical consultation?	Yes / No<br><br><br>Yes / No	Yes
Did you attend the emergency department since the last time that we talked?	No / Yes, [reason]	Yes
Did you have any hospitalization since the last time that we talked?	No / Yes, [reason]	Yes
Is there anything else you want to tell me?	No / Yes, [Free answer]	

Questions during Follow-up phone calls (Acenocumarol/Warfarin)

LOLA's question	Patient's answer	Alert Criteria
Do you feel better, worst or the same as the last time we talked?	Same/ Better/ Worst	
Do you take your medication every day?	No / Yes	
Did you forget to take your medication in the last month?	No / Yes, [number of times] / Don't know	
Did your primary care physician made any change in your chronic medications?	No / Yes / Don't know / [Free answer]	Yes
Did you change your usual diet?	No / Yes / Don't know	Yes
Are you taken any new vitamin or herbal supplements?	No / Yes	
Regarding your anticoagulant treatment, do you know if your last TRT was under 65%?	No / Yes / Don't know	Yes/ Don't know

Did you have any bleedings in the last month?<br>If the answer is yes, do you feel you need a medical consultation?	No / Yes, [number of times]/ Don't know.<br><br>No/ Yes	Yes
Did you feel loss of strength, speaking difficulties or acute loss of vision in the last month?<br>If the answer is yes, did you seek medical attention?	No / Yes / Don't know.<br><br>Yes / No	Yes
Do you have any surgical intervention planned in the next month?<br>If the answer is yes, do you need a medical consultation?	Yes / No<br><br>Yes / No	Yes
Did you attend the emergency department since the last time that we talked?	No / Yes, [reason]	Yes
Did you have any hospitalization since the last time that we talked?	No / Yes, [reason]	Yes
Is there anything else you want to tell me?	No / Yes, [Free answer]	Yes
